# Prospective Clinical Evaluation of the Singularity™ Air Laryngeal Mask in Adult Patients

**DOI:** 10.3390/jcm12237312

**Published:** 2023-11-25

**Authors:** Joana Martins, Bernhard Beutel, Nadja Ettlin, Norbert Nickel, Roman Wüthrich, Roman Sandoz, Angel Borisov, JoEllen Welter, Alexander Dullenkopf

**Affiliations:** Spital Thurgau Frauenfeld, Institute for Anesthesia, Pfaffenholzstr. 4, 8501 Frauenfeld, Switzerland; joana.martins@kssg.ch (J.M.); bernhard.beutel@kssg.ch (B.B.); nadja.ettlin@stgag.ch (N.E.); norbert.nickel@stgag.ch (N.N.); roman.wuethrich@stgag.ch (R.W.); angel.borisov@paraplegie.ch (A.B.); joellen.welter@stgag.ch (J.W.)

**Keywords:** laryngeal mask, airway management, general anesthesia

## Abstract

A laryngeal mask is one of the most widely used airway management devices. The Singularity^TM^ Air is a second-generation laryngeal mask whose shaft angle can be adjusted after insertion. Since the device’s performance has been assessed on mannequins only, this study aimed to evaluate Singularity^TM^ Air’s effectiveness in the clinical setting. The prospective single-center cohort study included 100 adults undergoing elective surgery under general anesthesia and suitable for airway securing with a laryngeal mask. The primary endpoint was the oropharyngeal leak pressure, and the secondary endpoints were the ease of insertion and the patient’s comfort. Laryngeal mask insertion was successful in 97%, and mechanical ventilation was possible in 96% of patients. After insertion, the median (IQR) oropharyngeal leak pressure was 25 (18–25) cm H_2_O, which remained stable at 25 (25–25) cm H_2_O after 20 min. The median (IQR) time for successful manual bag ventilation was 42 (34–50) seconds. Nineteen patients complained of side effects (e.g., sore throat, difficulty swallowing), but none persisted. The Singularity^TM^ Air performed well in a clinical setting, and its oropharyngeal leak pressure was comparable to that of other masks reported in the literature. The time for successful manual ventilation was slightly longer, and patients reported more temporary side effects.

## 1. Introduction

Since its introduction in the early 1980s, the laryngeal mask has quickly became one of the most widely used airway management devices in anesthesia [1]. The laryngeal mask has been incorporated into guidelines for managing difficult airways, further reflecting its acceptance and contribution to the anesthesiology field [2,3]. Improvements to the original device resulted in a second generation of laryngeal masks with a mechanism for gastric drainage [4].

Many studies have been published comparing different laryngeal masks, including systematic reviews and meta-analyses [5,6]. In most studies, the parameters ‘ease of insertion’ and ‘airway seal (or leak)’ are the most commonly used quality indicators [7].

**Figure 1:**
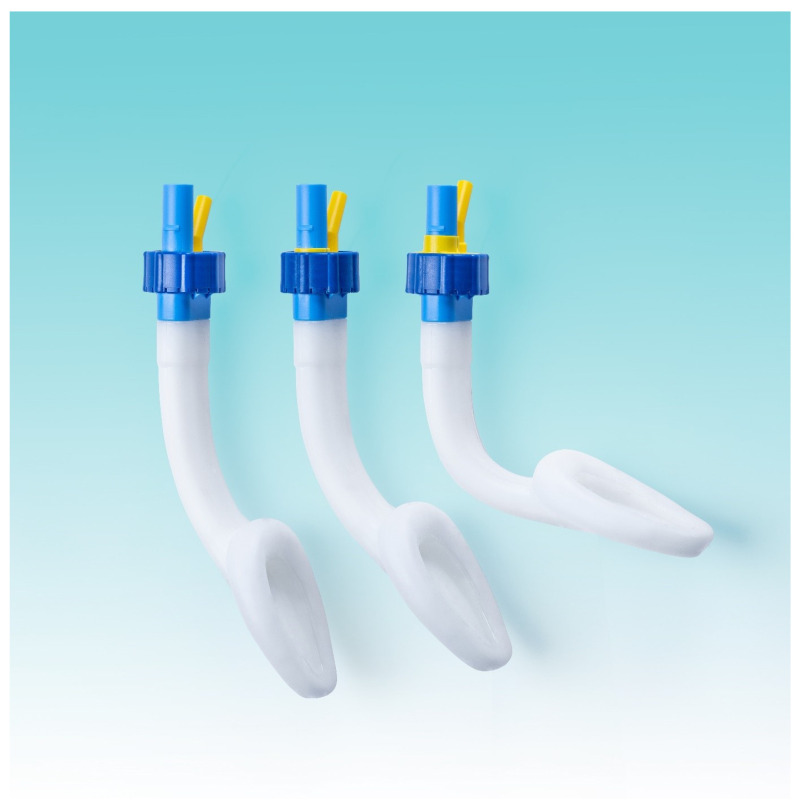
Laryngeal mask Singularity™ (Singularity AG, Maur/Zurich, Switzerland). Left: as delivered; Right: laryngeal mask shaft maximally bent.

The Singularity™ Air laryngeal mask (Singularity AG, Maur/Zurich, Switzerland; Figure 1) expands on the second generation’s design by including a dial to adjust the shaft angle after insertion, which ought to improve the fitting to the patient’s airway and thereby airway sealing. The Singularity™ Air laryngeal mask’s performance has been measured on mannequins only [8]. Our study’s primary aim was to evaluate the mask’s effectiveness under clinical conditions by measuring the oropharyngeal leak pressure, and the secondary endpoints were to determine the ease of insertion and patient comfort.

## 2. Materials and Methods

This prospective single-center study was carried out with the approval of the Ethics Committee of Eastern Switzerland (EKOS 21/191, 9 November 2021) and registered with German Clinical Trials Register (DRKS00029113). The study was conducted from January to June 2023 at a Swiss acute care hospital where approximately 9000 general anesthetics are performed annually.

Patients undergoing elective surgery under general anesthesia and determined to be suitable candidates for a laryngeal mask based on the judgment of the attending anesthesiologist were assessed for inclusion. The patient-based inclusion criteria were: American Society of Anesthesiologists (ASA) physical status I–III, fasting according to internal guidelines (6 h for solid food, clear liquid allowed up to anesthesia), and ≥18 years of age. The primary exclusion criterion was any contraindication to using a laryngeal mask, such as morbid obesity (body mass index ≥ 35 kg/m^2^ based on institutional standards) or symptomatic esophageal reflux disease. According to institutional guidelines, surgery in which the patient is not supine or is in the Trendelenburg position, laparoscopies, and ear–nose–throat surgery are unsuitable for airway securing by laryngeal masks. Further exclusion criteria were pregnancy, emergency surgery, loose teeth, inability to follow the investigation procedures (e.g., language problems, psychological disorders, dementia), participation in another study, or previous enrolment in the current investigation. All patients provided written informed consent to participate in the study.

### 2.1. Study Device

Singularity™ Air laryngeal masks are available in two adult sizes. According to the manufacturer’s instructions, size 4 is designed for patients weighing 70 kg or less, and size 5 is for patients > 70 kg. The laryngeal masks were prepared for use by emptying the cuff using a syringe, lubricating the outer curvature with a sterile gel (Instillagel; FARCO-PHARMA, Köln, Germany), and slightly bending the shaft.

### 2.2. Management of Anesthesia

The anesthesia induction was performed according to institutional guidelines. Patients were given 3.75 to 7.5 mg midazolam orally 30 min before being transferred to the operating room, where they received standard monitoring (i.e., non-invasive blood pressure measurement, electrocardiogram, and pulse oximetry using the IntelliVue MP30 vital sign monitoring (Philips, Zurich, Switzerland)), a peripheral venous cannula for the administration of fluids and medications, and connection to an EEG-based Bispectral Index (BIS) anesthetic monitor (Philips, Zurich, Switzerland). 

Before starting the anesthetic, patients were preoxygenated by tightly applying a ventilation mask to their face and administering oxygen up to an end-tidal fractionated oxygen concentration (FiO_2_) of 0.8 with the patient breathing independently. The anesthetic was administered as a propofol-based, total intravenous anesthesia (TIVA) with a target-controlled infusion (TCI) syringe pump based on the Schnider pharmacokinetic model (Alaris PK Syringe Pump, CareFusion, Rolle, Switzerland) that uses the patient’s age, weight, height, and gender. The standard setting for the anesthesia induction was a target effect-site propofol concentration (Cet) of 6 mcg/mL and infusion of remifentanil 0.1 mcg/kg/min. For the induction of anesthesia, 1–3 mcg/kg of fentanyl was administered intravenously. Before placement of the laryngeal mask, a BIS value of lower than 60 and the possibility of gentle bag–mask ventilation were required. After securing the airway, the propofol Cet was reduced to maintain a BIS value of between 40–60 while factoring in the patient’s blood pressure, heart rate, and general clinical observations. 

The exact time point for insertion of the laryngeal mask was determined by the responsible anesthesiologist. The laryngeal mask was inserted, as suggested by the manufacturer, with the patient’s head slightly extended. A second person assisted in opening the patient’s mouth by performing the Esmarch maneuver. If insertion was not possible on the first attempt, the following steps were taken: induce deeper anesthesia, change insertion technique (180° rotation, increase bending, guidance by finger), or use neuromuscular blocking drugs. After insertion of the laryngeal mask, its cuff was filled with air up to a cuff pressure of 60 cm H_2_O using a hand-operated cuff manometer (cuff manometer; VBM, Sulz am Neckar, Germany). If manual ventilation by bag was insufficient after insertion of the laryngeal mask, the remedial steps were as follows: induce deeper anesthesia, reposition the laryngeal mask, increase the bend in the tube, and use neuromuscular blocking drugs. If insertion or manual ventilation after insertion were still not possible, another airway device was used (institutional standard first-generation laryngeal mask or tracheal tube).

The patient was then mechanically ventilated (Atlan™ or Primus™; Dräger, Lübeck, Germany; tidal volume 6 mL/kg, frequency 8–12/min adjusted for appropriate end-tidal CO_2_, positive end-expiratory pressure (PEEP) 5 cm H_2_O, inspiratory oxygen concentration (FiO_2_) 0.6) according to institutional standards. If mechanical ventilation was deemed insufficient by the responsible anesthetist due to inadequate tidal volume or unreliable CO_2_-tracking, measures were taken to adapt ventilator parameters (e.g., reduce the PEEP, increase the inspiratory time, reduce the breathing frequency), and manipulate the laryngeal mask. A gastric tube (14 Ch; pfm medical, Köln, Germany), lubricated with sterile gel (Instillagel; FARCO-PHARMA, Köln, Germany), was advanced through the gastric lumen of the Singularity™ Air. Successful gastric placement of the probe was verified by auscultation over the stomach when insufflating air using a 50 mL syringe. At the end of surgery, emergence from general anesthesia and removal of the laryngeal mask were performed according to institutional guidelines.

The medical staff administering the anesthesia included senior doctors, experienced residents, and certified anesthesia nurses. All study team members were first trained on a mannequin and subsequently inserted the Singularity™ Air in patients at least five times before participating in this study.

### 2.3. Data Assessment

Oropharyngeal leak pressure, defined as the airway pressure up to which the laryngeal mask prevents insufflated air from escaping from the patient’s mouth, was assessed by increasing the airway pressure to the point that air leakage could be heard. To determine this point, the ventilator/manual bag switch of the anesthesia equipment was turned to manual ventilation. The adaptive pressure release valve (APV) of the circle circuit was then set to 40 cm H_2_O, and fresh gas flow was to 8 L/min. Airway pressure was increased by manually restricting the ventilation bag. Increasing airway pressure was limited to 25 cm H_2_O, as displayed on the anesthesia equipment monitor. This procedure was repeated after 15 to 30 min. 

We recorded the time from stopping bag–mask ventilation in order to start airway manipulation to the first successful manual ventilation by bag through the laryngeal mask. Successful ventilation was defined as thoracic–abdominal movement followed by an appropriate capnography signal. Any drop of the peripheral oxygen saturation (SpO_2_) to less than 90% was recorded. At the end of the anesthetic, the laryngeal mask was removed, and the tip was inspected for the presence of blood. The duration of the anesthetic was documented. 

On the first postoperative day, the patients were asked (in person if hospitalized or by telephone for outpatient cases) to rate throat soreness on a scale from 0 (no soreness at all) to 10 (worst sore throat imaginable). In addition, we asked patients if they were experiencing difficulties when swallowing or dysesthesia in the mouth or pharynx, and if they required treatment to alleviate symptoms. Patients were advised to contact the study team should any discomfort persist.

### 2.4. Statistical Analyses

Data are presented descriptively as mean and standard deviation or median and interquartile range based on normality tests (Shapiro–Wilk test). Comparisons between time points were performed using the Wilcoxon signed-rank test. A *p*-value of <0.05 was considered statistically significant. All analyses were conducted in Stata (version 15, StataCorp, College Station, TX, USA).

## 3. Results

Forty-one of the 100 patients included in the study were fitted with a size 4 laryngeal mask and the remaining 59 were fitted with a size 5. The patients’ ages ranged from 18 to 88 years. Study participants underwent the following surgical procedures: 31% orthopedic, 25% urologic, 22% visceral surgery, 20% gynecological, and 2% mixed procedures. Table 1 summarizes the demographic data of the entire study cohort and according to the mask size group.

At the initial assessment after laryngeal mask insertion, the median oropharyngeal leak pressure was 25 (IQR 18–25, range 5–25) cm H_2_O (size 4 = 25 (IQR 25–25, range 5–25) and size 5 = 24 (IQR 16–25, range 5–25)). The oropharyngeal leak pressure approximately 20 min after the first measurement improved for the entire cohort (*p* = 0.0003) (with the same median but a narrower interquartile range resulting from a higher minimum value) to 25 (IQR 25–25, range 15–25) cm H_2_O. These same values were observed for sizes 4 and 5, which also showed improvements (*p* = 0.0144 for size 4 and *p* = 0.0024 for size 5, respectively).

No difficulties occurred during insertion in 65% of the patients (58% and 69% in laryngeal mask sizes 4 and 5, respectively). For most of the remaining patients, insertion was successful after altering the insertion technique, such as guiding the laryngeal mask by finger or inserting a catheter in the gastric lumen (*n* = 15), bending the tube (*n* = 5), providing deeper sedation (*n* = 5) or neuromuscular relaxation (*n* = 1), making a second attempt (*n* = 5), or repositioning the patient’s head (*n* = 1). Insertion of the laryngeal mask was not successful in three patients (one with a laryngeal mask size 4 and two with size 5); therefore, an alternative airway device had to be used.

Manual bag ventilation through the laryngeal mask was successful in 92% of the patients. For five additional patients (*n* = 2 laryngeal mask size 4 and *n* = 3 size 5), the tube had to be bent (*n* = 3), the laryngeal mask had to be repositioned (*n* = 1), or deeper sedation was needed (*n* = 1). The median time required for successful manual bag ventilation through the laryngeal mask was 42 (IQR 34–50) seconds (44 (IQR 39–59) in size 4 and 40 (IQR 32–48) in size 5 laryngeal masks). Oxygen saturation did not drop below 90% for any patients before bag ventilation through the laryngeal mask was established.

Mechanical ventilation succeeded in 78 of the 97 patients (80%). In an additional 18 patients (20%), bending the laryngeal mask’s tube or modifying the respirator settings was required to facilitate mechanical ventilation. Only one patient needed an alternative airway device. Table 2 presents the results of mechanical ventilation and gastric tube insertion in the 97 patients with successful laryngeal mask insertion. A gastric tube was successfully inserted through the intended channel in 91 patients. In four of these patients (all with a laryngeal mask size 5), manipulation of the laryngeal mask (bending (*n* = 3) or applying Esmarch maneuver (*n* = 1)) was needed to insert the tube. In six patients, it was not possible to insert a gastric tube (4 (10%) with laryngeal mask size 4 and two (5%) with size 5). 

The median duration of anesthesia was 70 (IQR 58–93) minutes. When removing the laryngeal mask, the tip was stained with blood in 23% of the cases (30% of size 4 and 18% of size 5 laryngeal masks). 

On the first day after surgery, seven patients complained of a sore throat that they rated above three on a ten-point verbal analog scale (2 (5%) with size 4 and 5 (8%) with size 5 laryngeal masks). Nine patients reported difficulties swallowing after anesthesia (4 (10%) with size 4 and 5 (8%) with size 5 laryngeal masks). Pharyngeal dysesthesia after anesthesia was reported by three patients (2 (5%) with size 4 and 1 (2%) with a size 5 laryngeal mask). None of these complications required therapy, and none of the patients reported back because of persistent discomfort after hospital discharge or after the telephone call on postoperative day one.

## 4. Discussion

Laryngeal masks are important tools for airway management in anesthesia and emergency medicine [2,3]. This study evaluated the recently introduced laryngeal mask, Singularity™ Air, in a routine clinical setting. The overall success rate of the mask was 96%. The median oropharyngeal leak pressure, assessed by auscultation and restricted to a maximum of 25 cm H_2_O, was well established at the time of insertion and its effectiveness further improved slightly 20 min after insertion.

Given the high insertion and ventilation success rates of laryngeal masks in general, leak pressure is a more meaningful parameter to measure [1,7,9]. Compared to the gold standard of airway management, tracheal intubation by an endotracheal tube, laryngeal masks are considered less reliable for sealing the airway. Different methods for assessing leak pressure are reported in the literature [10]. The measurement technique used in this study, oropharyngeal leak pressure, is easy to perform and interpret. Once the increasing airway pressure exceeds the oropharyngeal leak pressure provided by the laryngeal mask, air escaping from the patient’s mouth is heard. The airway pressure cannot be set to deliberately high values for a long time, as the set pressure eventually generates positive end-expiratory pressure (PEEP) that could harm the patient’s cardiovascular system. Therefore, we established 25 cm H_2_O as our maximum pressure. This is a clinically meaningful threshold, given that peak inspiratory pressure is often set to a maximum of 20 cm H_2_O for mechanical ventilation. Even though the leak per se would not be a reason to change a laryngeal mask, a prolonged leak of a certain amount would eventually result in unreliable respiratory monitoring, unreliable tidal volumes, and (if administered) leakage of inhalational anesthetic agents. Our study cohort had a similar median leak pressure of 25 cm H_2_O after insertion and after 20 min. However, the distribution shifted upwards and the range of values narrowed, resulting in statistically significant improvements in the leak pressure. It is possible that the warming of the laryngeal mask causes some material softening, which results in a better adjustment to the pharyngeal structures. These outcomes were comparable to other studies reporting similar values [6,11,12,13,14,15,16,17]. Since ventilation was performed with PEEP set to 5 cm H_2_O in our study, the leaking pressure may have been affected [18]. Although we did not conduct systematic assessments, ventilation improved for some patients after measures such as modifying respirator settings or altering the bend of the device’s shaft.

We report a relatively long time-to-adequate ventilation compared to other studies. In some studies, this time was only 4 s when using another laryngeal mask (for which, however, others reported 44 s [6,17]. Although time-to-insertion is frequently reported as a surrogate for the ease of handling the laryngeal mask, it is often not clearly defined. Unfortunately, a standard for assessing this parameter has not yet been established. In our study, we began tracking insertion time when bag–mask ventilation stopped. Therefore, the number of seconds needed for handling of anesthesia mask and breathing circuit components, proper head positioning, opening the patient’s mouth, and reaching for/applying the laryngeal mask were included in the insertion time. Perhaps a more important topic than the potentially inconsistent assessment methods is the question of the clinical relevance of this parameter. As a matter of safety during elective anesthesia, the difference between 20 or 60 s after adequate pre-oxygenation and bag–mask ventilation is inconsequential. Our study population’s peripheral oxygen saturation never dropped below 90% before the airway was secured.

Although the time-to-successful ventilation was within the range of other devices when, for example, using a guided insertion technique or for military novices inserting laryngeal masks, the Singularity™ Air’s design presents some challenges. Gasteiger et al. attributed the difficulty of inserting the Singularity™ Air to the soft tip of the device, resulting in a predetermined kinking point [8]. In that mannequin study, the overall success rate for insertion and sufficient ventilation was 92%, slightly lower than our findings with human patients. Their study’s primary endpoint, time-to-successful ventilation, was a mean of 24 s. Our median value was 42 s. One further possible explanation for this discrepancy is that properly positioning a patient’s head and adjusting the opening of the mouth may require more time than necessary for a mannequin.

An alternative explanation could be related to the stiffness of the tube. Therefore, insertion techniques and tips, such as to first position the device against the hard palate and advance it caudally by pushing initially ‘up’ against the palate—which keeps the device posterior and tends to avoid dragging the tongue down into an obstructing position—may be more difficult to perform. The extra stiffness could stem from the built-in mechanism that makes the manual adjustment of the tube bending possible. Using lubrication gel on both sides of the device could help overcome this. Other possible explanations or contributing factors may be that our study population did not receive neuromuscular blocking agents before laryngeal mask insertion, and we did not exclude toothless patients.

With the first-generation laryngeal masks, insertion of a gastric tube is not possible, as it is with second-generation devices or in tracheal intubation. The success rate reported in the literature for gastric tube insertion in second-generation devices varies. We inserted the tube in 90% of the patients in whom ventilation by the Singularity™ Air was successful, which is in the lower range of other published studies. The gastric tube we used in this study had a diameter of 14 Ch (approximately 4.7 mm), which is not possible in some second-generation laryngeal masks (for example, i-gel™ supraglottic airway; intersurgical, Sankt Augustin, Germany). A gastric tube with a larger diameter may be advantageous when gastric content other than air needs to be removed.

After removal, blood at the tip of the laryngeal mask is a sign of injury, caused mainly by the insertion of the airway device. While blood on the tip is a commonly reported outcome, it has not been directly linked or correlated with patient discomfort. Our rate was within the range of other published studies, but our patient population reported higher values for side effects such as a sore throat, pain when swallowing, and hoarseness. It is worth noting that our patients did not receive neuromuscular blockers. Fortunately, none of these complaints required therapy or persisted. As previously mentioned, the relative stiffness of the tube may have contributed to these outcomes.

Our study had some limitations that ought to be mentioned. First, there was no strict protocol followed for managing cases of insufficient leak pressure. As a result, the effectiveness of bending the shaft of the laryngeal mask Singularity™ Air under these conditions remains unclear. The same applies to the effect of bending the shaft during gastric tube insertion or for tracheal intubation through the device. Second, while a study population of 100 is within the range of other studies comparing devices and evaluating leaking pressures, it may be inadequate for detecting safety issues. Third, although this study was designed as a descriptive clinical evaluation of a new device, using a comparison group would have been advantageous and should be the next step for further studies. Lastly, the size of the laryngeal masks was selected depending on the patients’ weight. This is what the manufacturer suggests, but in reality, it may be advantageous to select the size depending on height and/or gender.

In conclusion, the recently introduced laryngeal mask Singularity™ Air performed relatively well in a clinical setting, but may not have outperformed the earlier generation or other second-generation devices. Since we did not compare the device to other laryngeal masks, further studies may be warranted. Specifically, investigations should focus on (1) closely assessing the functioning of the unique rotary wheel (dial), its ability to effectively bend the device after insertion, and its impact on improving airway sealing; (2) evaluating the possibility of tracheal intubation through the Singularity™ Air; and (3) selecting the appropriate size of the device.

## Figures and Tables

**Table 1:** Demographic patient and anesthesia data according to laryngeal mask size.

		Total	LM* Size 4	LM* Size 5
Patients	*n*	100	41	59
Sex	female	53	36 (88)	17 (36)
Age	years	46.2 (17.0)	47.7 (18.3)	45.2 (16.1)
Height	meters	1.72 (0.1)	1.66 (0.1)	1.76 (0.1)
Weight	kilograms	74.9 (14.2)	62.4 (7.5)	84.0 (10.6)
Body mass index ^†^	kg/m^2^	24.7 (22.3–27.3)	22.7 (20.8–24.2)	26.3 (24.6–30.2)
ASA status	I–III	2.0 (0.5)	2.0 (0.5)	2.0 (0.5)
Mallampati score	I–IV	1.6 (0.8)	1.7 (0.8)	1.5 (0.7)
Denture	yes	6	4 (10)	2 (3)

Data are *n* (%), mean (standard deviation) or ^†^ median (interquartile range); LM* = laryngeal mask.

**Table 2:** Mechanical ventilation and gastric tube insertion in patients after successful insertion of laryngeal mask.

	Total (*n* = 97)	LM* Size 4 (*n* = 40)	LM* Size 5 (*n* = 57)
Mechanical ventilation successful without additional measures	78 (80)	35 (88)	43 (75)
Measures to facilitate mechanical ventilation	19 (20)	5 (12); lengthening inspiration (*n* = 3), reducing PEEP† (*n* = 1), LM removal (*n* = 1)	14 (25); Lengthening inspiration (*n* = 4), reducing PEEP† (*n* = 6), bending shaft (*n* = 3), neuromuscular blockade (*n* = 1)
Mechanical ventilation not successful	1 (1)	1 (2.5)	0
Insertion of gastric tube successful	87 (90)	36 (90)	51 (89)

Data are *n* (%). LM* = laryngeal mask; PEEP† = positive end-expiratory pressure.

## Data Availability

The data presented in this study are available on request from the corresponding author. All obtained data are presented in the manuscript.
